# Persistence of distinctive morphotypes in the native range of the CITES‐listed Aldabra giant tortoise

**DOI:** 10.1002/ece3.1764

**Published:** 2015-11-07

**Authors:** Lindsay A. Turnbull, Arpat Ozgul, Wilna Accouche, Rich Baxter, Lindsay ChongSeng, Jock C. Currie, Naomi Doak, Dennis M. Hansen, Pierre Pistorius, Heather Richards, Janske van de Crommenacker, Rainer von Brandis, Frauke Fleischer‐Dogley, Nancy Bunbury

**Affiliations:** ^1^ Department of Plant SciencesUniversity of OxfordSouth Parks RoadOxfordOX1 3RBU.K; ^2^ Institute of Evolutionary Biology and Environmental StudiesUniversity of ZurichZurichCH‐8057Switzerland; ^3^ Seychelles Islands FoundationLa Ciotat BuildingMont FleuriVictoriaMahéSeychelles; ^4^ South African Environmental Observation NetworkCape TownSouth Africa

**Keywords:** Aldabra giant tortoise, allopatric variation, morphological variation, population dynamics, spatial subdivision

## Abstract

Understanding the extent of morphological variation in the wild population of Aldabra giant tortoises is important for conservation, as morphological variation in captive populations has been interpreted as evidence for lingering genes from extinct tortoise lineages. If true, this could impact reintroduction programmes in the region. The population of giant tortoises on Aldabra Atoll is subdivided and distributed around several islands. Although pronounced morphological variation was recorded in the late 1960s, it was thought to be a temporary phenomenon. Early researchers also raised concerns over the future of the population, which was perceived to have exceeded its carrying capacity. We analyzed monthly monitoring data from 12 transects spanning a recent 15‐year period (1998–2012) during which animals from four subpopulations were counted, measured, and sexed. In addition, we analyzed survival data from individuals first tagged during the early 1970s. The population is stable with no sign of significant decline. Subpopulations differ in density, but these differences are mostly due to differences in the prevailing vegetation type. However, subpopulations differ greatly in both the size of animals and the degree of sexual dimorphism. Comparisons with historical data reveal that phenotypic differences among the subpopulations of tortoises on Aldabra have been apparent for the last 50 years with no sign of diminishing. We conclude that the giant tortoise population on Aldabra is subject to varying ecological selection pressures, giving rise to stable morphotypes in discrete subpopulations. We suggest therefore that (1) the presence of morphological differences among captive Aldabra tortoises does not alone provide convincing evidence of genes from other extinct species; and (2) Aldabra serves as an important example of how conservation and management in situ can add to the scientific value of populations and perhaps enable them to better adapt to future ecological pressures.

## Introduction

Morphological variation in the Galapagos giant tortoise is said to have inspired Darwin's theory of evolution by natural selection. Darwin was told that the people of Galapagos could assign island of origin to any tortoise because of differences in the size and shape of the shell. Darwin attributed these differences to adaptive changes resulting from the varied ecological conditions that prevailed among islands. Today, molecular techniques have revealed that at least some of this variation has a genetic basis (Beheregaray et al. 2003; Caccone et al. 2004; Chiari et al. 2009), and likely reflects adaptations to the variety of soils and microclimates that force animals to use different types of food (Ciofi et al. 2009). The existence of these morphotypes, several of which have subspecies status, has had conservation implications, with captive breeding programs aimed at maintaining and restoring the ancestral diversity (Benavides et al. 2012).

**Figure 1:**
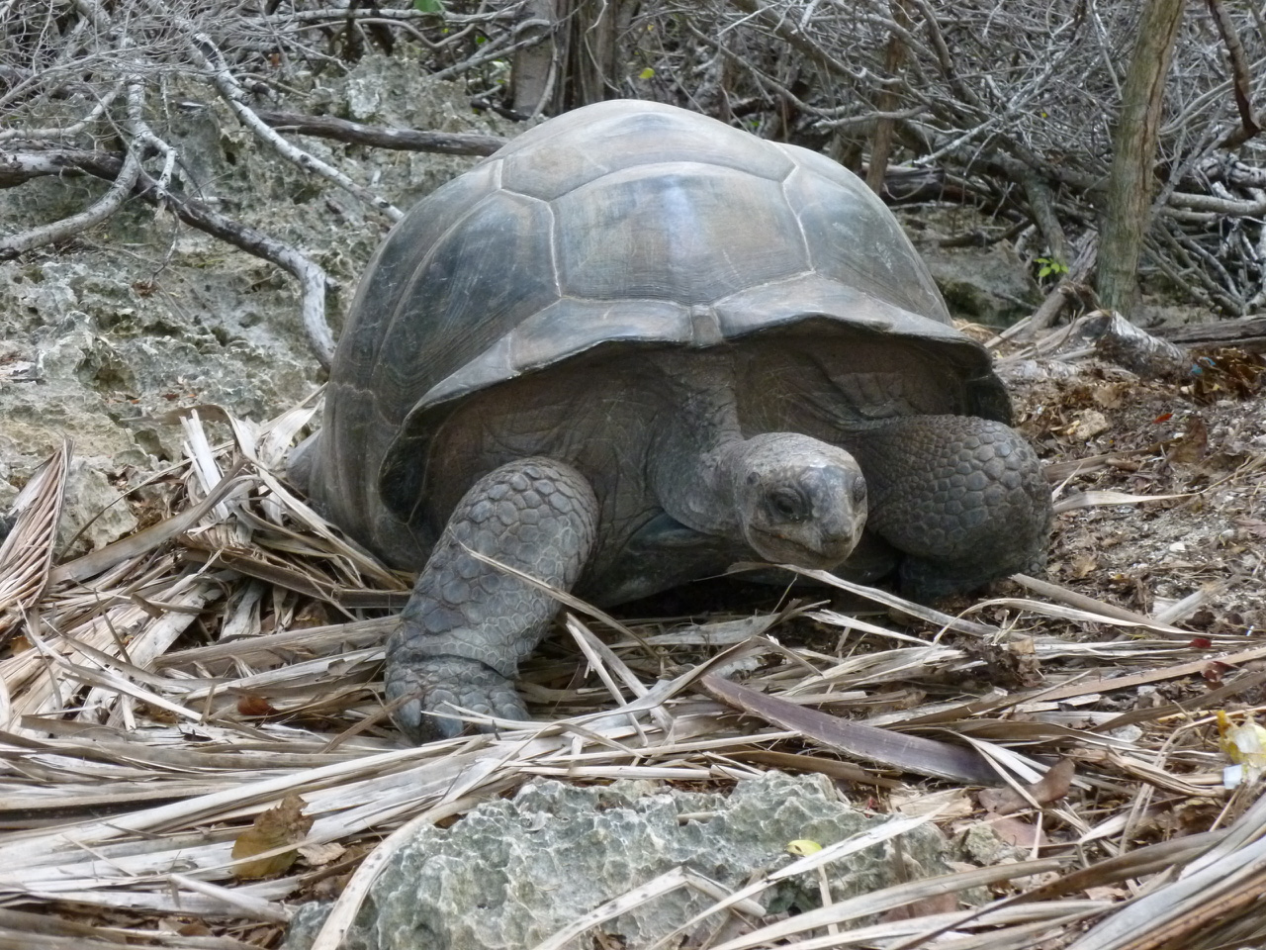
An adult Aldabra tortoise searches for food around the research station on Picard.

**Figure 2:**
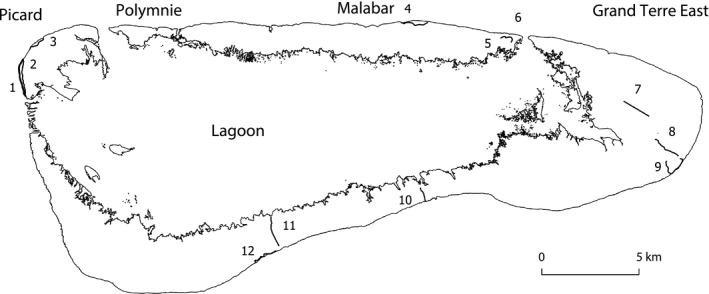
A map of Aldabra atoll showing main islands and subpopulations and the location of the twelve transects.

The giant tortoises of Galapagos, despite their fame, are not the most abundant free‐living species of giant tortoise alive today. That distinction belongs to the Aldabra giant tortoise (*Aldabrachelys gigantea* (Schweigger 1812); Fig. 1), the sole survivor of a radiation of Indian Ocean giant tortoises that once occupied many islands in the region (Palkovacs et al. 2002; Austin et al. 2003). The tortoise gets its name from Aldabra Atoll (Fig. 2), an isolated and remote island far‐removed from major shipping routes. The species is thought to number somewhere in the region of 100,000 animals (Bourn et al. 1999), in comparison with an estimated 19,000 free‐living tortoises on Galapagos (Márquez et al. 2004).

Aldabra, unlike Galapagos, is not an archipelago; however, the four main islands comprising the atoll – Grand Terre, Malabar, Picard, and Polymnie – are separated by deep‐water channels. The larger islands are further subdivided by areas of deeply fissured and dissected limestone, which tortoises cannot easily cross. This subdivision means that the atoll is home to several subpopulations of tortoises. The limited genetic analysis completed to date found variation in nuclear (although not mitochondrial) markers (Balmer et al. 2011), despite the human intervention that has moved animals around the atoll as part of an exploitation program that stretches back to at least the 18th century (Stoddart and Peake 1979). This raises the possibility that the Aldabra tortoise population was originally differentiated into geographically isolated morphotypes or even subspecies. This has conservation implications, as the Aldabra tortoise is often used in restoration projects (Griffiths et al. 2011) and there is controversy over whether morphological variation in captive populations is due to the presence of genes from other Indian Ocean tortoise species, now extinct (Palkovacs et al. 2003; Gerlach 2009).

Early authors from a Royal Society initiative commented on the remarkable variation in body size and sexual dimorphism observed in tortoises around the atoll (Gaymer 1968; Grubb 1971; Merton et al. 1976). But they unanimously assumed that this variation was temporary and due only to variation in density, which they believed was due to differences in historical exploitation pressure (Gaymer 1968; Grubb 1971; Merton et al. 1976). For example, on Grand Terre (Fig. 2) tortoises appeared to be at high density, particularly in the southeast, and these animals were small with little evidence of sexual dimorphism (Hnatiuk et al. 1976; Merton et al. 1976; Bourn and Coe 1978). In contrast, animals on the more accessible island of Malabar (Fig. 2) were larger with pronounced sexual dimorphism (Grubb 1971; Bourn and Coe 1978). The theory – that observed differences in the characteristics of subpopulations around the atoll were temporary and due to variations in the history of exploitation – was entirely plausible at the time. The theory was backed up by further investigations, which seemed consistent with these ideas. For example, female tortoises in the southeast of the atoll laid fewer, smaller eggs than females on Malabar (Swingland and Coe 1978, 1979), and animals from Grand Terre grew faster when translocated to Picard (Merton et al. 1976; Bourn and Coe 1978).

To test the prediction that subpopulations would converge in density a census was conducted in 1997 (Bourn et al. 1999) and compared with data from a first census taken in 1973–1974 (Bourn and Coe 1978). However, the two datasets are not ideal for such a comparison, as the censuses were conducted at different times of year (tortoise movements are highly seasonal; (Swingland and Lessells 1979; Gibson and Hamilton 1983)) and used different methodologies. To account for these differences, adjustments were made to the later census data, which are not clearly specified, so it is difficult to assess their suitability. However, the resulting comparison concluded that differences in density among the subpopulations, although still apparent, were substantially reduced (Bourn et al. 1999). This convergence was suggested to be in part due to a population “crash” in the southeast, apparently confirming earlier fears about tortoises damaging their environment (Bourn and Coe 1978) and raising concerns about the long‐term viability of the giant tortoise population. As a direct result of this analysis, a more detailed long‐term monthly monitoring program was established in January 1998, which continues to the present day.

Here, we analyze data from a recent 15‐year monitoring period (January 1998 to December 2012) to test: (1) whether there is evidence for recent changes in population size; (2) whether tortoise density differs among subpopulations; (3) whether differences in size and degree of sexual dimorphism persist or whether there has been convergence in morphological traits among subpopulations; and (4) whether subpopulations differ in their sex ratios. Additionally, we use data from a subsample of marked individuals to investigate rates of population turnover.

## Materials and Methods

### Aldabra atoll

Aldabra is a UNESCO World Heritage Site in the Western Indian Ocean (9°24′S 46°20′E) with a land area of 155.4 km² and an average height above sea level of ~ 8 m. The atoll consists of a fragmented ring of islands encircling a large lagoon (Fig. 2). The four main islands are Grand Terre (116.1 km²), Malabar (26.8 km²), Picard (9.4 km²), and Polymnie (4.75 km²) (Fig. 2). The land is covered in either thick coastal scrub – mainly *Pemphis acidula* (Forst.) – or a mixture of trees, herbs, and grasses, including species‐rich grasslands known as tortoise turf (Gibson and Phillipson 1983a). The atoll is home to the world's largest remaining population of giant tortoises, the Aldabra giant tortoise, *Aldabrachelys gigantea*, which appears on CITES appendix 2. The island is managed by the SIF (Seychelles Islands Foundation) who maintain a permanent research base on Picard and conduct regular monitoring.

The climate on Aldabra shows pronounced seasonal variation (Fig. S1). Higher rainfall is linked to the northwest monsoon, which occurs from December to April, while August is the driest month. Depending on the exact duration and timing of the monsoon, annual precipitation patterns may vary considerably. Further, most exceptionally wet years correspond to positive IOD (Indian Ocean Dipole) events on Aldabra. Seasonal fluctuations in rainfall drive seasonal variation in the availability of food. Tortoises respond to these fluctuations in their food supply through seasonal movements, which can be pronounced, particularly on Grand Terre (Swingland and Lessells 1979; Gibson and Hamilton 1983).

### Data collection

We analyzed a database of monthly population counts collected over a period of 15 years: January 1998 to December 2012, which is publically available via Dryad. Monthly counts were made on 12 transects distributed around the atoll on three of the four main islands: Grand Terre, Picard, and Malabar (there is no extant population of tortoises on Polymnie, although it may have had tortoises in the past). The largest island, Grand Terre, has a central area of deeply dissected and fissured limestone, considered difficult for tortoises to traverse. In all previous studies, it has therefore been treated as having two subpopulations: Grand Terre East and Grand Terre West, although we acknowledge that the real extent of tortoise movements between these two areas is unknown. Transects were deliberately located in a range of vegetation types and are not, therefore, an unbiased sample of the island as a whole. There are three transects in each of the four subpopulations (Fig. 2). Transects are of variable length with a width of 10 m, except on Picard where one transect (Coastal) is 20 m wide, and are subdivided into 50 m‐long sections (Table S1). Thus, sections consist of a sampling area of either 500 m^2^ or 1000 m^2^. To remove differences in the area sampled, we analyzed section densities (i.e., counts per unit area) rather than the raw counts. To remove seasonal effects and better reveal long‐term trends, we averaged across months and analyzed mean densities in each section in each year.

A vegetation type is assigned to each section on each transect based on a vegetation map produced by Gibson and Philipson (1983b). Seven major types of vegetation are identified; these are Pool and Mangrove; Coastal Scrub; Coastal Grassland; Standard Mixed Scrub; Open Mixed Scrub; *Pemphis* Scrub; and *Casuarina* and Coconut Palm. These vegetation types are not evenly distributed among islands; for example, *Casuarina* and Coconut Palm only occurs on Picard and Malabar. Hence, vegetation type and subpopulation are not orthogonal.

During monthly monitoring, the size and sex of each individual encountered on transects are recorded. The size of animals is recorded by measuring the greatest width across the third vertebral scute of the carapace. This measurement was established in the 1960s and is very highly correlated with carapace length (*r* ^*2*^ = 0.98; Grubb (1971)), which in turn is highly correlated with body mass (Aworer and Ramchurn 2003). There is no fully objective method for sexing animals, although they differ in the size and shape of the carapace and plastron and the length of the tail and hind claws, and large animals can usually be sexed reliably. However, the features used for sexing are less well‐developed in smaller animals; hence, individuals with a third‐scute measurement <20 cm are usually, but not always, recorded in the database as “sex unknown”. To be conservative, we assigned all those animals with a third‐scute measurement <20 cm as “sex unknown”. When examining trends through time, we analyzed males, females, and unknowns separately.

Most animals encountered on transects are unmarked; however, during the time of the Royal Society Expedition to Aldabra, numbered titanium disks were attached to the carapace of some animals on Grand Terre and Malabar. Disks were attached to 700 tortoises by Gaymer in 1969 and 1970 (Gaymer 1973), while a further 6182 tortoises were marked in the same way by Bourne in 1973–1974 (Coe et al. 1979). Animals with these disks can still be found on the atoll, and they are noted, with their number, when they appear on transects. We used this data to estimate relative survival rates of males versus females in the different subpopulations.

### Statistical analysis

To quantify the spatial distribution of tortoises and trends through time, we analyzed: (1) occupancy (presence/absence of tortoises) on all sections; and (2) mean densities in occupied sections (i.e., those sections containing at least one tortoise in at least 1 month of the year). Analyses were carried out using generalized linear mixed‐effects models within the statistical package R version 3.1.0 (R Core Team 2014). We accounted for the spatial structure in the data in the random effects: sections are nested within transects, which in turn are nested within subpopulations. For the occupancy (presence/absence) analysis, we used a generalized linear mixed‐effects model with a binomial error structure; for the mean densities in occupied sections, we applied a log transformation to the data (this produced excellent residual plots). To test whether population sizes have changed through time we fitted year, subpopulation and their interaction as fixed effects. We considered models of all animals collectively, and only analyzed males, females and unknowns separately where there was a significant overall trend. Retention of terms was judged by comparing AIC values associated with different models.

The body size of individuals (third‐scute measurements) was analyzed using linear mixed‐effects models (the function lmer) with the same random effects structure as specified above. Residual plots revealed that no transformation was necessary. To test whether males and females differ in size among subpopulations, we fitted sex, subpopulation, and their interaction as fixed effects. Retention of terms was judged by comparing AIC values associated with different models, and all estimates (with their standard errors) are from the minimal adequate model in each case. Because the same individuals are likely to be encountered and measured repeatedly, we also performed an analysis for each year separately. To investigate the possibility of changes in the size of animals through time, we also considered models using the whole data set in which the fixed effects included year and its interactions with sex and subpopulation. Finally, we analyzed the sex ratios on sections, again using generalized linear mixed‐effects models (glmer) with a binomial error structure. The functions lmer and glmer are within the R package lme4 (Bates et al. 2014).

Survival of tagged individuals was analyzed using a mark–recapture model implemented using Program MARK (White and Burnham 1999) and RMark interface (Laake and Rexstad 2010). Because transect counts were performed monthly and we are interested in annual survival, we pooled the re‐sighting data annually and estimated apparent survival and recapture rates using the Cormack–Jolly–Seber model (Lebreton et al. 1992). We used data only from Grand Terre East, Grand Terre West, and Malabar, because no individuals were marked on Picard. In total, we used data from 166 females, 98 males, and 63 individuals of unidentified sex. Using the length of the third scute as a time‐varying individual covariate, we tested for the effect of body size on survival while taking into account variation among islands and sexes. We applied a goodness‐of‐fit test using RELEASE TEST 2 + 3 to test whether our global model fits the data. Because this dataset is considerably smaller than the count dataset, we used Akaike's information criterion corrected for small sample size (AIC_c_) for the identification of the most parsimonious model (Burnham and Anderson 2002). Based on the estimated annual survival rates and their standard deviation, we bootstrapped survival rates for 40 consecutive years, the cumulative product of which gave the resulting fraction of initially marked adults alive after 40 years for each sex and island.

## Results

### Population size and trends through time

**Figure 3:**
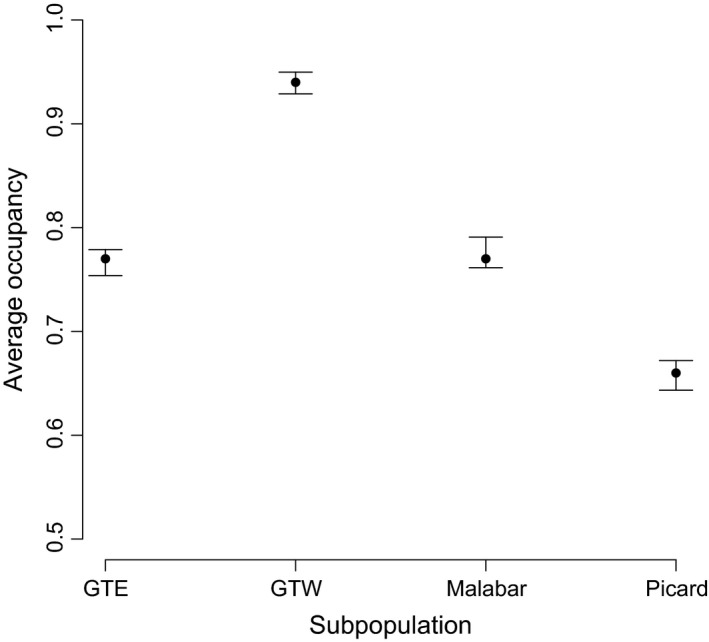
Differences in the average occupancy of 50 m transect sections among subpopulations. A section is defined as occupied if at least one tortoise was found in that section in at least 1 month of the year.

The occupancy of sections – that is, the probability that a section has at least one tortoise in at least 1 month in the year, is generally high in all subpopulations, with the highest occupancy on Grand Terre West (0.94; Fig. 3). Occupancies in the other three subpopulations were lower (Grand Terre East = 0.77; Malabar = 0.78; Picard = 0.66; Fig. 3). There was evidence of a very slight increase in occupancy during the monitoring period on both Grand Terre East (slope = 0.0433; SE = 0.00869) and Malabar (slope = 0.0247; SE = 0.0106), while on Grand Terre West and Picard, the trend is also increasing, although nonsignificant. On Grand Terre East occupancies of males, females, and unknowns all increased, although only males and unknowns increased significantly (♀s: slope = 0.0162; SE = 0.0161; ♂s: slope = 0.0867; SE = 0.0143; unknowns: slope = 0.0821; SE = 0.0333) On Malabar male and female occupancies did not show significant trends (♀s: slope = −0.0335; SE = 0.0250; ♂s: slope = 0.0371; SE = 0.0214) while the unknowns showed a significant increase (slope = 0.0531; SE = 0.0166). This indicates an increase in the number of smaller animals on Malabar. The inclusion of vegetation type had a modest effect on the AIC (a decrease of around nine units), and inspection of parameter estimates indicates that tortoise occupancies are highest in Coastal Scrub and Open Mixed Scrub, and these vegetation types are commonest on Grand Terre.

The best model for average density in occupied sections included both vegetation type and an interaction between subpopulation and year. The addition of subpopulation to the best model resulted in an increase in the AIC of four units. An alternative model which included the main effect of subpopulation rather than vegetation type produced an AIC value that was 21 units higher. Hence, it seems that differences in density among subpopulations are mostly due to differences in vegetation type. Densities were highest in Coastal Scrub, Coastal Grassland, and Open Mixed Scrub. The direction and magnitude of changes in density through time differ among subpopulations but are very small in all cases. The mean density has significantly increased on Grand Terre West (slope = 0.0309; SE = 0.00468) and on Picard (slope = 0.0130; SE = 0.00320). On Grand Terre East and Malabar, there has been no significant change. However, even on Grand Terre West, which has the highest estimated slope, the predicted increase in density over the fifteen‐year monitoring period is very small.

### Size and sexual dimorphism

**Figure 4:**
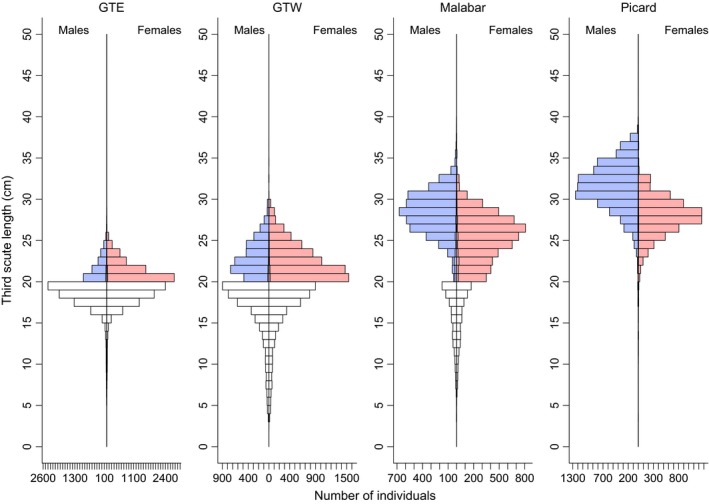
Histograms of third‐scute measurements of males and females in each of the four subpopulations (GTE = Grand Terre East; GTW = Grand Terre West). Unshaded bars indicate animals of unknown sex, which have been equally assigned to males and females.

The size of animals differs dramatically among subpopulations (Fig. 4). These average size differences are accompanied by large differences in the degree of sexual dimorphism: for example, on Grand Terre East and West, females are small and males are only slightly larger than females, while on Malabar and Picard, females are larger, but males are much larger than females (Fig. 4); that is, where the average size is large, the degree of sexual dimorphism is more pronounced. The inclusion of this interaction between subpopulation and sex received strong statistical support (ΔAIC = 8575). When models were fitted to each of the 15 years separately, the model including the interaction between subpopulation and sex was always preferred (ΔAIC = 380–950 units).

The average size of animals has changed through time, and these changes differ among islands (inclusion of the interaction caused a decrease in AIC of 295.4 units). Inspection of parameter estimates shows that the average size of animals has decreased slightly on Grand Terre West (slope = −0.0203; SE = 0.00661) and on Malabar (slope = −0.0780; SE = 0.00809), while the average size of animals has increased slightly on Picard (slope = 0.0988; SE = 0.00697). The trend on Grand Terre East was also negative but nonsignificant. Given that animals are currently largest on Picard and smallest on Grand Terre these trends are inconsistent with the idea that animals from different populations are converging in size. When we analyzed trends for each sex separately, we found that male and female sizes followed the same pattern: that is, both males and females declined in size on Grand Terre East, Grand Terre West, and Malabar, while both males and females increased in size on Picard. However, we emphasize that the changes in size through time are tiny compared to the average size differences among subpopulations.

### Sex ratios

Figure 4 reveals marked differences in the sex ratios of larger animals (scute‐width >20 cm) among subpopulations. Analysis of the proportion of females (among animals of confirmed sex) confirms this impression. There are significant differences in the proportion of females among islands, with a female‐biased sex ratio on Grand Terre East (P(♀) = 0.724) and Grand Terre West (P(♀) = 0.655), while the sex ratio is close to 1:1 on Malabar (P(♀) = 0.552) and on Picard (P(♀) = 0.510). These apparent differences in the sex ratio are correlated with the degree of sexual dimorphism: Hence, where males are clearly larger than females, the sex ratio is close to 1:1, whereas when females and males are of similar size, the sex ratio appears to be female biased.

There is evidence that the sex ratios have changed through time and the magnitude of this change differs among subpopulations (the model which included the interaction between subpopulation and year had an AIC value 70 units lower than the model with only a main effect of year and 147 units lower than a model with no year effect). Populations have become less female‐biased on both Grand Terre East (slope = −0.0424; SE = 0.00436) and Picard (slope = −0.0262; SE = 0.00342).

### Size and apparent survival

In the survival analysis, the goodness‐of‐fit test provided no evidence for lack of fit (*χ* ^2^_201_ = 183.2, *P* = 0.81); hence, we did not correct for over‐dispersion in the data. The candidate model set is shown in Table S2. The most parsimonious model provided evidence for: (1) an interaction between subpopulation and sex on recapture rates; and (2) a main effect of subpopulation and an interaction between size and sex on apparent survival rates. Recapture rates ranged between 0.19 ± 0.04 (Grand Terre West ♂s) and 0.45 ± 0.04 (Malabar ♀s) (Fig. S2a). Annual survival rates ranged between 0.82 ± 0.03 (Malabar ♂s) and 0.95 ± 0.02 (Grand Terre West ♂s) (Fig. S2b). The effect of body size on survival was significant and negative only in males on Malabar (slope: −0.46 ± 0.14); larger males on this island were therefore more likely to die compared to smaller ones (Fig. S3). Based on these of annual survival rates, the estimated fraction of initially marked adults alive after 40 years was less than 7% for all sex and island combinations except for males in GTW (15.3% ± 0.05).

## Discussion

The giant tortoise population on Aldabra Atoll is the only surviving intact population of a group of herbivores that once dominated many of the islands in the Indian Ocean and beyond (Arnold 1979; Hansen et al. 2010). It is therefore of particular conservation relevance. Concern had been raised from earlier studies that the population on the largest island, Grand Terre, was in decline as a result of overgrazing (Hnatiuk et al. 1976; Merton et al. 1976). Our analysis reveals that fears about rapid declines in the population are currently groundless. The population has been stable over the last 15 years, with only slight changes in both occupancy and density on most transects. Importantly, there is no evidence for substantial declines in any of the four monitored subpopulations.

Early observers noted dramatic differences in the body size of animals around the atoll and attributed this variation to differences in density, which they believed reflected variation in historical hunting pressures (Grubb 1971). However, our analysis reveals that current differences in density among subpopulations are more likely due to differences in vegetation type. There is therefore no evidence to support the idea that subpopulations will converge in density and phenotypic characteristics. In contrast, size differences among populations noted in the 1960s have persisted to the present day and show no sign of diminishing. Phenotypic differences have even increased over the last 15 years: Animals have decreased slightly in size on Grand Terre (despite the recent eradication of competing feral goats), while they have continued to increase in size on Picard.

There is inevitably some concern that the size differences among subpopulations might be caused by a sampling artifact, as transects are not randomly located. In other words, without knowing the atoll better it is reasonable to suggest that there are in fact many large animals on Grand Terre and many small animals on Picard, but they are simply not found on transects. However, researchers and rangers regularly patrol the atoll and are confident that the size differences observed on transects are representative of the subpopulations more generally. Large animals are never found on Grand Terre, and small animals are rarely encountered on Picard. Further, when small animals are found on Picard, they are clearly juveniles.

The size difference is intriguing, and the cause is unclear. One possibility is that all populations are food limited, but that differences in the quality and type of vegetation or the availability of fresh water causes differences in growth rates or survival among juveniles (Grubb 1971). A second possibility is that populations on Malabar and Picard are recruitment‐limited and have much lower rates of population turnover. This could mean that very few juveniles enter the population, reducing competition for food among survivors and allowing them to grow larger and enjoy a longer lifespan. Early authors and current observations support the idea that nest site availability is highly restricted on both Malabar and Picard, and that females are prone to digging up each other's eggs (Swingland and Coe 1978). The predator communities also differ among islands which could cause differences in recruitment rates: For example, Grand Terre is still home to feral cats, which are absent from both Malabar and Picard. Cats rarely predate tortoises, but their presence has eliminated the flightless, endemic Aldabra rail (*Dryolimnas cuvieri aldabranus* Pucheran) from Grand Terre. This bird certainly attacks and kills young tortoises and may be a cause of recruitment limitation on Malabar and Picard. However, while just about plausible for Picard, small tortoises are reasonably frequent (and indeed increasing) on Malabar (Fig. 4), which makes explanations based on recruitment limitation rather unlikely. In addition, survival rates of marked animals were lowest on Malabar and highest on Grand Terre West, which is inconsistent with the idea that animals from subpopulations with large body size are longer lived.

The substantial divergence in morphology among the subpopulations of tortoises on Aldabra could be plastic or genetic, or a combination of the two. Indeed, it may be that the ready plasticity in size and shape facilitates evolution and genetic differentiation (Price et al. 2003). However, the observed morphological variation appears to have been stable over 50 years. While these are long‐lived animals, our survival analyses indicate that mortality rates are high enough that we are not simply measuring the same animals as were measured 50 years ago. The differences are almost certainly caused by differences in ecology: the terrain, vegetation, predator community, and access to fresh water all differ among the islands. This variation could also account for morphological differences among captive Aldabra tortoises on the granitic Seychelles, previously attributed to lingering genes from tortoise lineages long thought to be extinct (Palkovacs et al. 2003; Gerlach 2009). A more detailed analysis of the genetics of the indigenous Aldabra tortoise population should now be a conservation priority.

The differences in sex ratio are intriguing. However, we acknowledge that there are uncertainties in sexing animals. In the future, an unequivocal method of determining sex (such as a portable ultrasound machine) would be extremely useful to assess the reliability of current sexing methods. The strong female bias on Grand Terre transects, where the dominant vegetation type is tortoise turf, may reflect differential habitat use by males and females. For example, perhaps these transects are particularly valuable to female tortoises who may have higher nitrogen (or other mineral) requirements associated with egg laying.

This analysis reveals the value of whole ecosystem conservation. The population of giant tortoises on the atoll is not in fact a single population, but a varied assembly of subpopulations exhibiting marked morphological divergence conserved in situ. It therefore offers a unique opportunity to better understand the processes of ecological and genetic specialization that led ultimately, for example, to the greater diversity of subspecies seen on Galapagos.

## Conflict of Interest

We have no competing interests.

## Data Accessibility

The tortoise database is available from Dryad: http://datadryad.org/submit?journalID=ECE3&manu=ECE-2015-03-00178.R1

## Supporting information


**Figure S1.** Seasonal variation in rainfall at the research station on Picard, Aldabra.
**Figure S2.** Recapture and survival rates of marked animals on Grand Terre East (gte), Grand Terre West (gtw) and Malabar (mal).
**Figure S3.** Size‐dependence of survival.

**Table S1**. Transect details.

**Table S2.** Mark‐recapture analysis of apparent survival rates.
