# Supplementary material for: Persistence of distinctive morphotypes in the native range of the CITES‐listed Aldabra giant tortoise
Source: Ecol Evol. 2015 Nov 7;5(23):5499–508. doi: 10.1002/ece3.1764 (PMC4813117; doi:10.1002/ece3.1764)
Supplement: Supplementary file 1 — Figure S1. Seasonal variation in rainfall at the research station on Picard, Aldabra. Figure S2. Recapture and survival rates of marked animals on Grand Terre East (gte), Grand Terre West (gtw) and Malabar (mal). Figure S3. Size‐dependence of survival. [file ECE3-5-5499-s001.docx]

**Supporting Information** for: *Sympatric subpopulations of endemic Aldabra giant tortoises have distinctive stable morphotypes***.**

**Figure S1.** Seasonal variation in rainfall at the research station on Picard, Aldabra. Black points are averages over the entire dataset. The grey points show rainfall recorded in individual years. Data is available from 1949 to 2012 with some gaps.

**
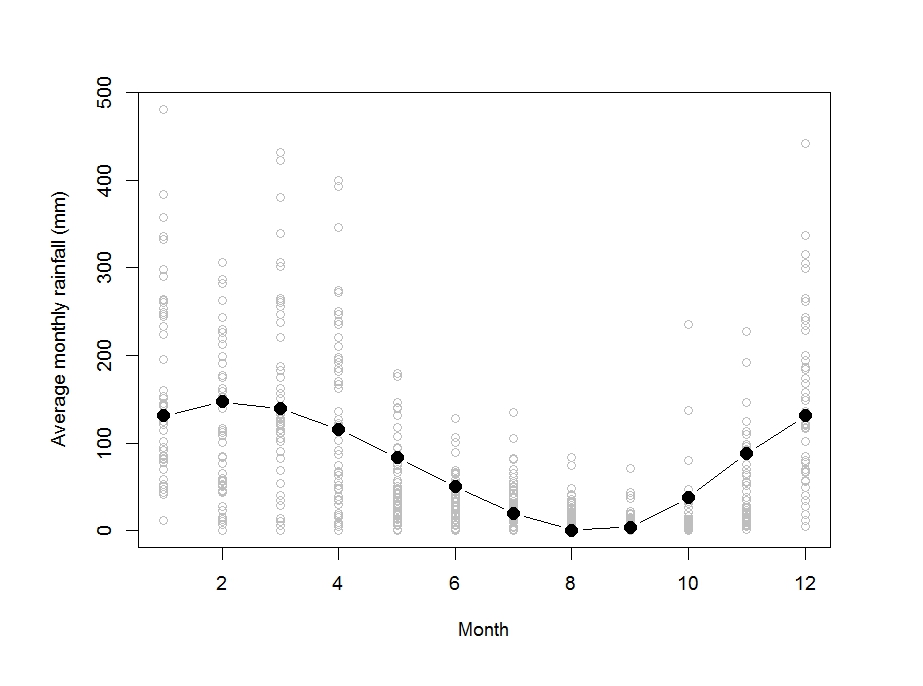
**

**Figure S2.** Recapture and survival rates of marked animals on Grand Terre East (gte), Grand Terre West (gtw) and Malabar (mal). Intervals are 95% confidence intervals.

**Figure S3. Size-dependence of survival.** Survival rates of males and females as a function of third-scute length in the three subpopulations. ‘Rugs’ show the 95% confidence interval in the regression slope.
