# Supplementary material for: Persistence of distinctive morphotypes in the native range of the CITES‐listed Aldabra giant tortoise
Source: Ecol Evol. 2015 Nov 7;5(23):5499–508. doi: 10.1002/ece3.1764 (PMC4813117; doi:10.1002/ece3.1764)
Supplement: Supplementary file 2 — Table S1. Transect details. [file ECE3-5-5499-s002.docx]

**Table S1: transect details.**

| **Number** | **Name** | **Subpopulation** | **No. of sections** | **Width (m)** |
| --- | --- | --- | --- | --- |
| 1 | Coastal | Picard | 40 | 20 |
| 2 | Back Path | Picard | 40 | 10 |
| 3 | Anse Var | Picard | 20 | 10 |
| 4 | Anse Malabar | Malabar | 30 | 10 |
| 5 | Passe Houareau Inland | Malabar | 20 | 10 |
| 6 | Passe Houareau Coastal | Malabar | 20 | 10 |
| 7 | Groves | Grand Terre East | 30 | 10 |
| 8 | Coco | Grand Terre East | 30 | 10 |
| 9 | Southern | Grand Terre East | 38 | 10 |
| 10 | Dune Jean Loius | Grand Terre West | 14 | 10 |
| 11 | Dune de Messe Inland | Grand Terre West | 32 | 10 |
| 12 | Dune de Messe Coastal | Grand Terre West | 30 | 10 |
