# Supplementary material for: Persistence of distinctive morphotypes in the native range of the CITES‐listed Aldabra giant tortoise
Source: Ecol Evol. 2015 Nov 7;5(23):5499–508. doi: 10.1002/ece3.1764 (PMC4813117; doi:10.1002/ece3.1764)
Supplement: Supplementary file 3 — Table S2. Mark‐recapture analysis of apparent survival rates. [file ECE3-5-5499-s003.docx]

**Table S2. Mark-recapture analysis of apparent survival rates**. Akaike’s information criterion corrected for small sample size (ΔAIC_c_), model AIC_c_ weights, number of parameters and model deviance for the Cormack-Jolly-Seber models fitted to the annual resighting data. Only the models with AIC weights are shown; third is the width of the third scute and is our measurement of animal size.

| **Apparent survival model** | **Recapture model** | **n** | **ΔAICc** | **Weight** | **Deviance** |
| --- | --- | --- | --- | --- | --- |
| third + subpop + sex + third:sex | subpop + sex + subpop:sex | 17 | 0.0 | 0.735 | 2729.1 |
| third + subpop + sex + third:subpop + third:sex | subpop + sex + subpop:sex | 19 | 2.6 | 0.203 | 2727.5 |
| third + subpop + sex + third:sex + subpop:sex | subpop + sex + subpop:sex | 21 | 5.7 | 0.043 | 2726.4 |
| third + subpop + sex + third:subpop + third:sex +subpop:sex | subpop + sex + subpop:sex | 23 | 7.4 | 0.018 | 2723.9 |
| third + subpop + sex + subpop:sex | subpop + sex + subpop:sex | 18 | 13.9 | 0.001 | 2740.9 |
